# Whole Gut Motility Patterns in Patients with Chronic Nausea and Vomiting

**DOI:** 10.3390/jcm13237127

**Published:** 2024-11-25

**Authors:** Mette W. Klinge, Anne Mette Haase, Nanna Sutter Rolighed, Klaus Krogh, Mark Scott, Vincent Schlageter, Esben Bolvig Mark, Gursharan Kaur Nandhra, Asbjørn Mohr Drewes, Anders Bergh Loedrup

**Affiliations:** 1 Department of Hepatology and Gastroenterology, Aarhus University Hospital, 8200 Aarhus N, Denmark; meteader@rm.dk (M.W.K.); nannactsr@gmail.com (N.S.R.); klaukrog@rm.dk (K.K.); 2 Blizard Institute, Queen Mary University of London, London E1 4NS, UK; m.scott@qmul.ac.uk; 3 Motilis Medica SA, 1007 Lausanne, Switzerland; vincent.schlageter@motilis.com; 4 Mech-Sense, Department of Gastroenterology and Hepatology, Aalborg University Hospital, 9000 Aalborg, Denmark; e.mark@rn.dk (E.B.M.); amd@rn.dk (A.M.D.); 5 Clinical Physics, Barts Health NHS Trust, The Royal London Hospital, London E1 1FR, UK; g.k.nandhra@qmul.ac.uk; 6 Department of Clinical Medicine, Aalborg University, 9220 Aalborg, Denmark; 7 Department of Medicine, Section of Gastroenterology and Hepatology, Goedstrup Hospital, 7400 Herning, Denmark; anloed@rm.dk

**Keywords:** gastrointestial motility, nausea, vomiting, transit times, dysmotility

## Abstract

**Background/Objectives:** Chronic nausea and vomiting (N/V) disorders are common in clinical practice. Our primary aim was to compare total and segmental gastrointestinal transit times as well as gastric contraction patterns in patients with chronic N/V syndrome to those of healthy volunteers (HVs). In the patient group, our secondary aim was to explore how symptoms and motility patterns were affected by a serotonin HT_4_ receptor agonist (Prucalopride). **Methods:** Patients with chronic N/V syndrome and HVs underwent baseline assessment of regional gastrointestinal (GI) motility/transit using the Motilis 3D-Transit system. Patients were then treated with Prucalopride 2 mg daily for 28 days, with the 3D-transit examination repeated within 10–20 days after treatment onset. Two self-administered questionnaires (the Gastrointestinal Symptom Rating Scale [GSRS] and Gastroparesis Cardinal Symptom Index [GCSI]) were used to assess patients’ symptoms. **Results:** A total of 19 patients (13 F; median age 25 years (IQR 22–39) and 55 HVs (25 F; median age 28 (24–35) were included. At baseline, no differences in regional GI transit times were found between groups. However, patients had a significantly lower gastric contraction amplitude than HVs (9 mmHg (IQR 8–11) vs. 12 (10–15: *p* < 0.001). In response to Prucalopride treatment, gastric emptying time was reduced from a median of 3.1 h to 1.6 h (*p* < 0.005). Further, the GCSI was significantly reduced from GCSI 3.0 (IQR 2.3–3.7) at baseline to GCSI 1.9 (IQR 1.3–3.2) with Prucalopride. **Conclusions:** Patients with chronic N/V syndrome have significantly lower gastric contraction amplitude than HVs and may symptomatically benefit from prokinetics. They do not, however, have evidence of panenteric dysmotility.

## 1. Introduction

Nausea and vomiting (N/V) are very common symptoms in clinical practice. According to the Rome Foundation, chronic N/V disorders include chronic N/V syndrome and the subtypes cannabinoid hyperemesis syndrome and cyclic vomiting syndrome [1]. Symptoms affect quality of life, increase the number of admissions to hospital and can be difficult to treat [2,3,4].

Chronic N/V syndrome is defined as bothersome nausea occurring at least 1 day per week and/or one or more vomiting episodes per week with no evidence of organic, systemic, metabolic or self-induced diseases explaining the symptoms. Chronic N/V shares clinical characteristics with the condition gastroparesis but lacks identifiable delayed gastric emptying. Gastroparesis is, contrary to chronic N/V syndrome, a relatively well described disorder. Prompted by current knowledge about gastroparesis, we aim to learn more about the pathophysiology underlying the chronic N/V syndrome.

Gastroparesis is usually defined as delayed gastric emptying without underlying obstruction. The gold-standard test for diagnosing gastroparesis is gastric emptying scintigraphy [5]. However, gastric emptying time is very variable both between and within individuals. Hence, patients diagnosed with gastroparesis based on delayed gastric emptying will often have a normal gastric emptying time when re-evaluated, in spite of ongoing symptoms [6]. Therefore, the distinction between gastroparesis and chronic N/V syndrome remains unclear, and in clinical practice, they are often treated as one.

Proposed pathophysiological mechanisms underlying gastroparesis include abnormal gastrointestinal motility, pyloric dysfunction, disordered gut–brain interaction, visceral hypersensitivity and autonomic neuropathy including inappropriate signaling of the vagal nerve, which otherwise plays a significant role in the regulation of nausea and vomiting [7,8,9,10,11,12,13]. Recent results from body surface gastric mapping with high-resolution electrophysiology indicate that differences in basic gastric contraction frequency and amplitude can help clarify pathophysiology in gastric motility disorders [14]. For example, high amplitude gastric electrical signals are typical of obstruction, while low amplitude signals indicate attenuated contractile force. Enteric autonomic neuropathy is troublesome to measure. Studies in diabetic patients often use cardiac autonomic neuropathy as a surrogate measurement of enteric neuropathy. Earlier studies found that autonomic neuropathy correlates with prolonged gastric emptying in diabetic patients [15,16]. The presence of autonomic neuropathy in patients with chronic N/V is, therefore, interesting to explore.

Chronic N/V disorders are commonly accompanied by symptoms that are likely derived from the small intestine or colon (i.e., constipation, early satiety, abdominal discomfort and bloating) [10,11]. Accordingly, delayed or rapid gastric emptying may be accompanied by abnormal transit times and abnormal contractility in both the small and large intestine [12,17]. Consequently, the diagnostic evaluation of gastroparesis and chronic N/V syndrome should incorporate assessment of the whole GI tract, with therapy directed in accordance with objective findings. Unfortunately, the understanding and treatment of chronic N/V disorders has been hampered by a paucity of methods providing valid and the highly detailed panenteric assessment of gastrointestinal motility.

Chronic N/V syndrome and gastroparesis are usually treated with prokinetics. Domperidone and Metoclopramide are often used, but unfortunately, both drugs can have serious side effects and their long-term use is restricted [18]. Erythromycin stimulates contractions of the stomach and small intestine. It is also commonly used for N/V disorders, but side effects are often seen and tachyphylaxis may develop [18]. Serotonin agonists (such as the 5HT_4_ agonist Prucalopride) are commonly used for chronic constipation and panenteric motility disorders. However, data on their use in chronic N/V syndrome are, to the best of our knowledge, not yet available [18].

For the present study, we hypothesized that patients with chronic N/V have gastric hypocontractility and prolonged small intestinal and colonic transit times. Further, we hypothesized that the stimulation of serotonin HT_4_ receptors in this patient group can reduce both segmental and total gastrointestinal transit times. Accordingly, the primary aim was to compare gastric emptying time and contraction patterns as well as small intestinal transit time and colonic transit times in healthy participants and in patients who had symptoms corresponding to the Rome IV criteria of chronic N/V syndrome. Our secondary aim was to explore how symptoms and motility patterns were affected by the stimulation of 5HT_4_ receptors using the agonist Prucalopride.

## 2. Materials and Methods

### 2.1. Participants

Patients were recruited from outpatient clinics at the Department of Hepatology and Gastroenterology, Aarhus University Hospital and Department of Internal Medicine, Goedstrup Hospital, Denmark between April 2018 and October 2021. All participants were at least 18 years of age.

To meet the ROME IV criteria for chronic N/V syndrome, patients had to suffer from recurrent nausea at least once per week or vomiting once or more per week for the last 3 months, with onset of symptoms at least 6 months earlier [1]. To be included in the study, patients had to have at least moderate symptoms according to the Gastroparesis Cardinal Symptoms Index (GCSI) [19,20]. Thus, all patients had a mean score > 2 for nausea/vomiting, postprandial fullness/early satiety, abdominal pain/discomfort, or bloating.

Exclusion criteria were explanatory pathological findings at upper endoscopy, pregnancy or breastfeeding, severe co-morbidity including vascular and renal disease, and disorders known to cause chronic N/V or gastrointestinal dysmotility (e.g., neurological disorders, diabetes, coeliac disease). Drugs known to affect gastrointestinal motility were paused at least one week prior to and during the study period to wash out the affect they may have had on GI motility.

A total of 55 healthy volunteers (HVs) with comparable age distribution were drawn from our previously published normative dataset to serve as a control group for comparison of gastrointestinal motility patterns and transit times [21].

The study was performed in accordance with the European Community of Good Clinical Practice and the Helsinki Declaration. The study was registered and approved by the Danish Health Authority (reference number M-2018-43-18) and the Middle Denmark Region Committee on Health Research Ethics (reference number 2018020368)

### 2.2. Study Timeline

After inclusion into the study, patients completed baseline questionnaires (GCSI and GSRS) and a baseline 3D-Transit examination. Within 1 month, they began 28 days of treatment with Prucalopride. A second 3D-Transit time examination was performed 10–20 days after initiation of treatment with Prucalopride, and follow-up questionnaires (GCSI and GSRS) were answered within a week following treatment.

### 2.3. Questionnaires

The following self-administered questionnaires were used to assess patient’s symptoms:

(1) The Gastroparesis Cardinal Symptom Index (GCSI): This is a validated 9-item questionnaire grading the severity of symptoms for the past two weeks using a 6-point Likert scale from 0 (none) to 5 (very severe). Scores are calculated as median or mean. In previous studies, the minimal clinical important difference in GCSI has been set to 0.73 regarding the symptoms of nausea, post-prandial fullness, bloating and excessive fullness [19,20].
(2) The Gastrointestinal Symptom Rating Scale (GSRS): a validated 15-item questionnaire that rates the severity of five symptom clusters: reflux, abdominal pain, indigestion, diarrhea, and constipation. It uses a 7-point Likert scale from 1 (none) to 7 (very severe) with total score and sub-scores calculated as median or mean. Reported minimal clinical important difference varies from 0.4 to 1 [22].

### 2.4. Motilis 3D-Transit System

The 3D-Transit system (Motilis Medica SA, Lausanne, Switzerland) is a validated, non-commercialized, minimally invasive system consisting of wireless electromagnetic capsules for ingestion, an extracorporeal, portable detector containing 4 sensors and visualization software [23,24,25,26]. Changes in position and orientation of the capsule during transit through the gastrointestinal tract reflect gastrointestinal contractile activity and progression dynamics. Detailed assessment of gastrointestinal contractility and transit patterns is, hence, achievable in a completely ambulatory setting. Several studies in recent years using this system have provided a deeper understanding of dysmotility in patients with various gastrointestinal disorders, e.g., ulcerative colitis, diabetic enteropathy and opioid-induced bowel dysfunction [21,26,27].

Protocol: After an overnight fast, study subjects were provided with a standardized meal and the detector was attached. Immediately afterwards, the capsule was swallowed. Following ingestion, subjects were instructed not to eat again for six hours to avoid prolonged gastric emptying. After 6 h, the content and time of meals were optional. Subjects were not allowed to work out during the experiment but, otherwise, they were able to keep up daily routines including work. Confirmation of retention of the capsules within or expulsion from the body was interrogated real-time during the study period. As soon as the capsule was expelled, the detector was removed, and the study was terminated.

Gastric emptying time was defined as the time from ingestion of the capsule to the transition from the stomach to duodenum. This transition was defined by the appearance of the duodenal arch and a change in contractility pattern from 3 contractions per minute in the stomach to 9–12 contractions per minute in the duodenum. Small intestinal transit time was defined as the time between pyloric passage and ileocaecal passage, determined as a sudden drop in contraction frequency to 3 contractions per minute. Total colorectal transit time was defined as the time interval between ileocaecal passage and exit of the capsule [26].

Every single contraction of the stomach was manually marked to calculate its amplitude and contraction frequency. The computation was performed using movement of the capsule and its rotation; hence, surrogate markers for gastric contraction amplitude were as follows: position amplitude, based on capsule movement in millimeters (mm), and rotation amplitude, based on capsule rotation around its own axis in degrees as previously described in detail [27].

Transit times and gastric contraction frequency and amplitude from HVs were used as normative data. The 95th percentile was used as the upper limit of normal for each segmental transit time (stomach, small intestine and colon).

### 2.5. Assessment of Vagal Tone

Inspired by several studies in patients with diabetes, we used cardiac autonomic neuropathy as a surrogate marker for autonomic neuropathy [15,16]. The earliest indicator of cardiac autonomic neuropathy is found during a deep breathing examination, while the Valsalva’s maneuver is affected by a greater degree of autonomic nerve impairment [28,29] Cardiac vagal tone was determined by examination of heart rate variability, a well-known proxy for cardiac autonomic neuropathy [30]. The CE labeled, non-invasive, handheld, commercially available device Vagus^TM^ (Medicus Engineering, Aarhus, Denmark) was used to determine heart rate variability during three conditions: (1) change from supine to upward position; (2) deep breathing; and (3) forceful expiration. A normal heart rate variability test has a score of 0; a score of 1 is interpreted as early cardiac autonomic neuropathy, and scores of 2 or 3 are categorized as manifest cardiac autonomic neuropathy [31].

### 2.6. Serotonin HT_4_ Agonist

Prucalopride (Resolor^®^, Takeda Pharmaceuticals International AG Ireland Branch, Ireland) is a selective, high-affinity, partial 5-Hydroxytryptamine-4 receptor (5-HT_4_) agonist shown to increase gut secretion and motility, the latter by exerting both gastro- and enterokinetic effects [32,33]. Unlike the less selective 5-HT_4_ agonist Cisapride, it is not related to cardiac toxicity [34]. Prucalopride was administered orally as tablets of 2 mg once a day for 28 days.

### 2.7. Statistics

Statistical analysis was performed using STATA version 16 (release 18. College Station, TX, USA: StataCorp LLC.), Microsoft Excel (Office 365) and Prism (version 9: GraphPad, Boston, MA, USA). Data are presented as medians and interquartile range. Differences in pre- and during-treatment transit times and symptoms were tested using Wilcoxon signed-rank test. Differences in transit times between HVs and patients were computed using Wilcoxon rank sum test. Correlation between gastric emptying and GCSI scores before and after Prucalopride were evaluated by the Spearman rank correlation coefficient. Level of statistical significance was set at *p* < 0.05. The 3D-Transit capsule has not previously been used to evaluate motility changes in patients with chronic nausea and vomiting. Therefore, a sample size could not be calculated.

## 3. Results

**Figure 1:**
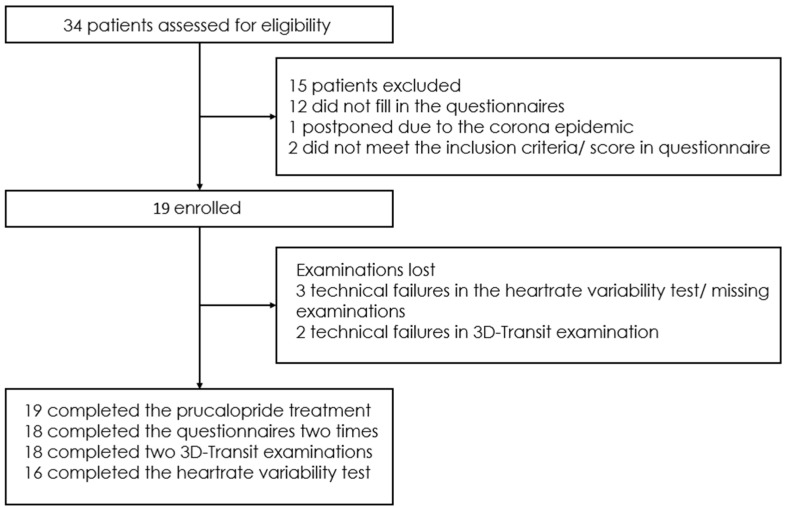
Trial flowchart.

Between 2018 and 2021, 34 patients with symptoms of chronic N/V syndrome were invited to participate in the study. Of these, 19 patients fulfilled the inclusion criteria and were willing to participate; 13 (68%) were female, median age (IQR) was 25 (22–39) and median BMI (IQR) was 21.3 kg/m^2^ (19.4–21.7). Reasons for not participating in the study are shown in Figure 1. The HV cohort comprised 55 individuals (44% female) with a median age (IQR) of 28 years (24–35) and a median body mass index (IQR) of 23.2 kg/m^2^ (21.6–25.8).

No serious adverse events were reported with regard to the 3D-Transit procedure or to the intake of Prucalopride. Mild and moderate adverse events related to start of Prucalopride were reported by six and two patients respectively; all were considered common transitory side effects (headache, nausea, loose stools, etc.). One patient stopped Prucalopride after 15 days of treatment because of side effects.

Patient characteristics, including the median GSRS and median GCSI and sub-scores at inclusion, are shown in Table 1. Heart rate variability was performed in 16 of 19 patients (one test failed due to technical issues and two were missing due to non-appearance). Although no patient had a definitive pathological cardiac autonomic neuropathy, nine (56%) had a borderline abnormal test.

### 3.1. Gastric Emptying and Gastrointestinal Transit Times

**Figure 2:**
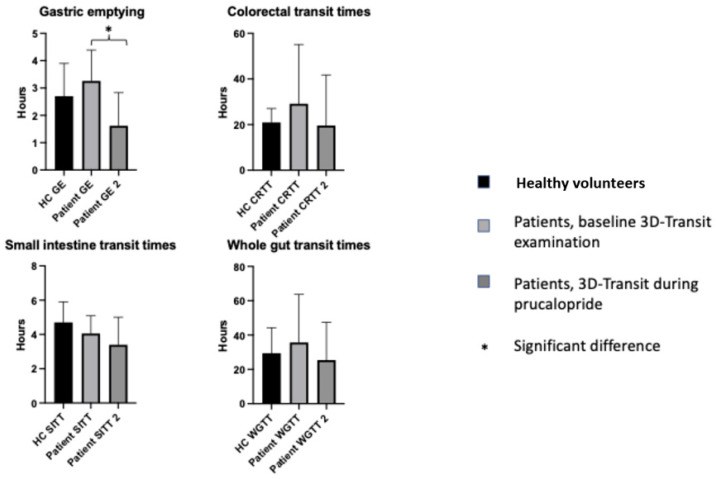
3D-Transit times. Data presented at medians; whiskers show interquartile range. * *p* < 0.05. Abbreviations: GE: gastric emptying, SITT: small intestinal transit time, CRTT: colorectal transit time, and WGTT: whole-gut transit time.

Group analysis showed that no differences in total or regional gastrointestinal transit times were found between patient’s baseline data and controls (Figure 2). However, using the upper 95th percentile from our group of HVs as the upper limit of normal, two patients (11%) were found to have a prolonged gastric emptying time, four patients (21%) had a prolonged colonic transit time and five patients (26%) had a prolonged total gastrointestinal transit time. No patient had a prolonged small intestinal transit time.

### 3.2. Gastric Contractions

Compared to HVs, the position amplitude of gastric contractions was lower in patients (median 9 mm [IQR: 8–11] vs. 12 [IQR: 10–15]; *p* < 0.001), but no difference was found for gastric rotation amplitude (median 34 degrees [IQR: 25–51] vs. 40 [IQR: 27–49]; *p* = 0.57), nor for frequency of gastric contractions (median 3.1 contractions per minute [IQR: 2.9–3.2] versus 3.0 [IQR: 2.9–3.2]; *p* = 0.21).

### 3.3. Effects of Stimulation of Serotonin HT_4_ Receptors

The median number of days (IQR) between starting on Prucalopride and the second transit time study was 13 (12–15). All 19 patients had a compliance rate to intake of Prucalopride of at least 80% before the second transit time study and all but two completed the full four weeks of treatment. Prucalopride significantly reduced gastric emptying time (IQR) from 3.1 h (2.1–4.4) to 1.6 h (1.3–2.8); *p* < 0.005. No group differences were found for small intestinal transit time, colonic transit time or total gastrointestinal transit time (Figure 2). For the patients with prolonged transit times at baseline, according to the HV upper 95% percentile (see above), all but one had a reduction in transit time for the affected bowel segment.

**Figure 3:**
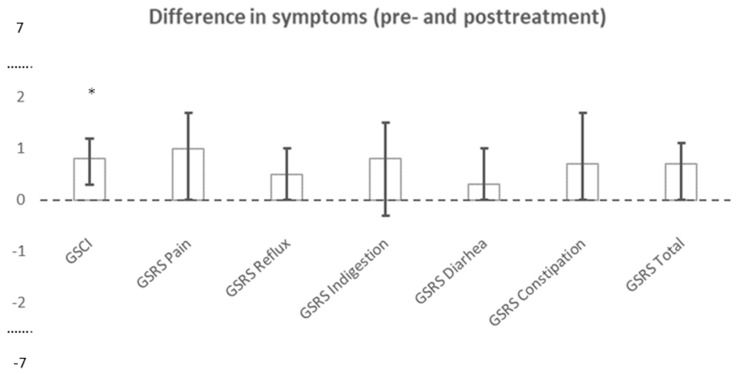
Difference in symptoms before and after treatment with Prucalopride. Data presented as medians with inter-quartile-range. GCSI: Gastroparesis Cardinal Symptom Index (range 0–5). GSRS: Gastrointestinal Symptom Rating Scale (range 1–7). * *p* < 0.05.

Following treatment with Prucalopride, a clinical difference of at least 0.73 in the GCSI was found in 13 of 19 patients (68%), and the median GCSI (IQR) changed from 3.0 (2.3–3.7) at baseline to 1.9 (1.3–3.2) post-treatment (*p* < 0.005) (Figure 3).

The median GCSI (IQR) was reduced from 3.0 (2.3–3.7) to 1.9 (1.3–3.2) (*p* < 0.05) during Prucalopride treatment, and the sub-score for nausea and vomiting was reduced from 2.3 (1.3–3.7) to 1.3 (1.0–2.3). A clinical important difference in GSRS sub-scores of at least 1 was most often seen for pain, which was relieved in 10 patients (53%), followed by constipation, which was relieved in eight patients (42%). Three patients (16%) experienced a worsening of symptoms related to constipation or diarrhea. An improvement in GCSI score was not associated with changes in gastric emptying (r_s_ = 0.33; *p* = 0.19); however, for the patients with prolonged transit time at baseline according to the HV upper 95% percentile (see above), all but one had a reduction in symptoms as recorded by the GSRS and GCSI.

## 4. Discussion

### 4.1. Main Findings

In the present study, we included 19 patients with significant upper gastrointestinal symptoms (GCSI > 2) who met the Rome IV criteria for chronic N/V syndrome. All patients also had moderate to severe symptoms assessed with the GSRS and most had symptoms indicating general intestinal dysmotility. However, when assessed with the 3D-transit system, only two patients had prolonged gastric emptying, four had prolonged colonic transit time and five had prolonged whole-gut transit time at baseline compared to healthy controls. The amplitude but not the frequency of gastric contractions was significantly reduced in patients compared to healthy participants. Gastric emptying time and symptoms of gastroparesis (GCSI) were reduced during Prucalopride treatment.

### 4.2. Gastrointestinal Transit Times

In our study population, group analysis showed that gastric emptying as assessed by the 3D-Transit system did not differ significantly from that of a population of healthy controls. Previously published data from wireless motility capsule studies show normative transit data similar to those presented here using the 3D-Transit system, indicating the reliability of our transit times [21,35,36]. In a recent study among healthy participants, gastric emptying time was shown to have wide limits of normal, varying from 20 to 240 min. The intraindividual coefficient of variation was 11% [37]. Also from previous studies, no strong correlation has been found between symptoms like nausea, vomiting, early satiety, postprandial fullness, abdominal discomfort and pain and gastric emptying time. Therefore, the clinical use of gastric emptying tests has been questioned [20,38,39].

When comparing patients to HVs at group level, we also found no difference between either small intestinal or colonic transit times. However, gastrointestinal motility disorders may, in some cases, be panenteric [40] and individual data showed that 11–26% of patients had either delayed regional or delayed total GI transit times at baseline. This reflects a heterogeneous group of patients but may also indicate panenteric dysmotility compromised by an under-powered study.

### 4.3. Gastric Motility Patterns

We found that patients with chronic N/V had a reduced amplitude of gastric contractions, but the basic frequency of contraction remained normal. This indicates that the underlying pathophysiology may be insufficient contractile force rather than obstruction or dysfunctional cells of Cajal. It is likely that reduced amplitude of contraction is a more subtle and reliable sign of poor gastric contraction force than prolonged gastric emptying time. The latter is defined by one point in time, namely, the exit of the capsule through the pylorus, while the median amplitude of gastric contractions is based on multiple contractions averaged over several minutes or hours. In patients with early autonomic neuropathy of the gut assessed with the wireless motility capsule, a low gastric motility index (mm Hg × sec/min) was shown to be associated with GI symptoms [41]. The pharmacodynamic effect of Prucalopride on gastric contraction amplitude is, to our knowledge, unknown. In the initial human studies, the effect of Prucalopride was determined by the number of colonic high-amplitude propagating contractions. In animal studies, Prucalopride was shown to enhance gastric emptying time.

### 4.4. Stimulation of Serotonin HT_4_ Receptors

Using Prucalopride, we attempted to change gastrointestinal motility patterns and assess associated symptom changes. Overall, small bowel, colonic and total gastrointestinal transit times did not change during treatment. However, gastric emptying and GCSI scores were both significantly reduced during treatment. Furthermore, one third of patients reached important symptom relief from symptoms of nausea, post-prandial fullness, bloating and excessive fullness. The majority of these patients initially reported symptoms of constipation before and relief of constipation during Prucalopride treatment, suggesting a correlation between upper gastrointestinal symptoms and colonic dysmotility.

We know from previous studies that Prucalopride enhances gastric emptying and stimulates colonic motility and, as such, it has a general effect on the gastrointestinal tract. Both mechanisms may contribute to symptom relief in our population [42], which exhibited symptoms of both constipation and gastroparesis. This is supported by the finding that in individual patients with prolonged transit time at baseline, all but one had a reduction in transit time for the affected bowel segment and symptom relief during treatment.

Abdominal pain was significantly reduced during treatment in 10 patients. Previously, abdominal pain has been reported in up to 90% of patients with gastroparesis, but no association between the severity of pain and gastric emptying time was found [43]. This may suggest underlying mechanisms causing abdominal pain other than slow transit or prolonged gastric emptying.

### 4.5. Cardiac Autonomic Neuropathy

No manifest cardiac autonomic neuropathy was recorded in the present study to explain gastrointestinal symptoms in this group of patients. However, nine patients (56%) had early signs or borderline cardiac autonomic neuropathy, with a score of 1 out of 3. The importance of this finding remains unknown, since more detailed heart rate variability test analysis was not performed.

### 4.6. Strength and Limitations

This prospective study was carried out in a real-life clinical setting using subjective patient-reported outcomes in combination with objective measurements. However, the study was observational in design and only included a small group of patients. To draw final conclusions, a larger cohort is needed. Future studies in larger cohorts of patients may be able to divide participants into subgroups according to pathophysiology since the group of patients with chronic N/V is very heterogeneous. Also, no long-term data on symptoms and gut motility are provided by this study.

The accuracy of the 3D-Transit system to detect clinical meaningful alterations in gut motility is comparable to that of other wireless motility capsule systems. This has been described in detail elsewhere [21,27]. Regarding specificity and sensitivity, we have no specific measures for the 3D-Transit system, but the wireless motility capsule (SmartPill) has a sensitivity of 59 to 86% and a specificity of 64 to 81% for gastroparesis [44].

The majority of included patients had undergone traditional determination of gastric emptying time before inclusion (most often scintigraphy), without revealing any significant findings. This is, however, not unusual in a non-diabetic population. Furthermore, the correlation between gastric emptying time and symptoms is known to be weak, and our patients had chronic troublesome symptoms, which made inclusion relevant despite normal findings on gastric emptying tests.

Patients paused drugs known to affect gastrointestinal motility at least one week prior to the study period but answered questionnaires regarding symptoms in the two weeks leading up to study entry. Therefore, baseline data cannot be clearly interpreted as the symptom burden for non-treated chronic nausea and vomiting. However, we do not believe this affected the results or the conclusion of this study regarding reduced gastric emptying time and reduction in GCSI following Prucalopride treatment.

Beside medication, diet is another important factor affecting the GI tract. We have no data regarding the diet used in the study population, and we cannot comment further on any possible impact this might have had on our results.

Furthermore, the placebo effect in medical treatment is well known and is also a potential bias in the current study. This should be taken into consideration when evaluating the questionnaires. Finally, there is a substantial day-to-day variation in gastrointestinal transit times, which influence the results of this study and require a larger study population to make more robust conclusions.

The groups of patients and HVs were not perfectly matched regarding age and gender. We acknowledge that both age and gender can affect gastric motility in healthy people (e.g., lower gastric contraction frequency in males, higher rotation amplitude in females, and a decrease in position amplitude with age) [27]. Based on our previous study, we consider the possible effect of gender differences on rotation amplitude and contraction frequency in the present study as insignificant [27]. Further, since neither gender nor age affect GE and a precise match between groups would mean a much smaller group of HVs, we did not match the groups one to one.

## 5. Conclusions

Patients in the present study, meeting the Rome IV criteria for chronic N/V syndrome, had a significantly lower position amplitude of gastric contractions at baseline compared to HVs. The patients are, however, not characterized as having panenteric dysmotility and they do not have established autonomic neuropathy as a cause of their symptoms. GE was reduced and they benefited symptomatically when treated with a serotoninergic prokinetic agent. Further research including a larger group of patients is required to confirm the results. 

## Figures and Tables

**Table 1:** Patient characteristics. Data are given in medians (IQR).

	Patients	Healthy Volunteers
Participants (n)	19	55
Age, yrs	25 (22–39)	28 (24–35)
Female gender, n (%)	13 (68)	25 (45)
BMI, kg/m^2^	21.3 (19.4–21.7)	23.2 (21.6–25.8)
Borderline vagal tone test, n (%)	9 (56)	
Baseline GCSI range 0–5	3.0 (2.3–3.7)	
Baseline GSRS range 1–7		
Pain	4.3 (3.7–5.7)	
Reflux	3.0 (2.0–4.0)	
Ingestion	4.3 (3.5–5.3)	
Diarrhea	2.3 (1.7–4.3)	
Constipation	3.3 (1.7–4.7)	
Total	3.5 (2.7–4.5)	
Medication, n	10	
Opioids	1	
Proton pump inhibitor	1	
Tricyclic antidepressants	0	
Antiemetics	7	
Laxative, osmotic	2	
Laxative, prokinetic agents	1	

GCSI: Gastroparesis Cardinal Symptom Index; GSRS: Gastrointestinal Symptom Rating Scale.

## Data Availability

The original contributions presented in the study are included in the article, further inquiries can be directed to the corresponding author/s.
